# Interview With Michael Bevan: The *Arabidopsis* Genome is Sequenced, What Next?

**DOI:** 10.1002/cfg.76

**Published:** 2001-04

## Abstract

Professor Michael Bevan is at the John Innes Centre, Norwich, UK, where he is Head of the Cell and Developmental Biology Department. He was the co-ordinator of the European Union *Arabidopsis* Genome Sequencing Consortium, which contributed to the sequence and analysis of the complete *Arabidopsis* genome that was published late last year. He is the co-ordinator of the EC funded EXOTIC program and is also a member of GARNet and UK CropNet. (For more background detail on these projects, please see our Featured Organism: *Arabidopsis thaliana* piece, also in this issue of CFG, or visit the websites listed below).


**Comparative and Functional Genomics**
*Comp Funct Genom* 2001; **2**: 99–102.
**DOI**: 10.1002/cfg.76

**Feature**

### Interview with Michael Bevan: The *Arabidopsis* genome is sequenced, what next?

### Abstract

**Professor Michael Bevan is at the John Innes Centre, Norwich, UK, where he is Head of the Cell and Developmental Biology Department. He was the co-ordinator of the European Union *Arabidopsis* Genome Sequencing Consortium, which contributed to the sequence and analysis of the complete *Arabidopsis* genome that was published late last year. He is the co-ordinator of the EC funded EXOTIC program and is also a member of GARNet and UK CropNet. (For more background detail on these projects, please see our Featured Organism: *Arabidopsis thaliana* piece, also in this issue of CFG, or visit the websites listed below).**

**CFG: Your group at the John Innes Centre works on the development of tools, such as GUS reporter systems and in vivo footprinting techniques: Do you see ways that these can be adapted, or applied in a global sense due to the knowledge of the sequence?**

MB: There are several potential applications of these methods to systematic analysis of the *Arabidopsis* genome. One is the use of the GUS reporter gene in gene trap constructs. For example, in Rob Martienssen's and my lab we use a modified Ds transposon that makes in-frame fusions with GUS upon insertion in a gene (Sundaresan *et al.* 1995). Large populations of these gene traps are being generated to reveal the spatial and temporal patterns of gene expression. This will complement array analyses, which generally do not reveal the cellular specificity of gene expression. I don't see *in vivo* footprinting being applied on a large scale, as it is very difficult. But chromatin immunoprecipitation, especially allied to array analysis, can reveal binding sites of transcription factors. Our ability to interpret the sequence information of promoters is very under-developed. But *Arabidopsis*, with essentially contiguous sequence, has nearly all of the potential regulatory sequences available for analysis. New bioinformatics techniques can be used to associate patterns of gene expression with promoter sequences, and establish likelihoods of particular sequences being associated with particular expression patterns. Here the availability of array data and gene trap data permits these approaches to be explored.

**CFG: You have an interest in characterising transcription factors and their binding sites, the sequence offers a further tool for the study of upstream regions of genes, do you plan to capitalise on this? Are there any projects moving in this direction?**

MB: There are opportunities to develop some prototypes for searching for promoter motifs in the "Exploiting Genomics" initiative from the BBSRC, but I don't know of any work in this field in *Arabidopsis* apart from the work of Maleck *et al.* ( 2000) who defined the WRKY element as a conserved functional feature in SA regulated genes, using cluster analysis of microarray data. This is clearly a field ripe for further development.

**CFG: The sequence also offers an unparalleled opportunity for comparative studies, which form part of your UK CropNet project, what are your plans in this area? How far do you think *Arabidopsis* data can be carried? [evidence for some synteny with tomato and soybean has been reported (Ku *et al.*, 2000; Grant *et al.*, 2000)**

MB: Comparative studies are very important at the John Innes Centre. The general goal is to identify crop plant orthologs of *Arabidopsis* genes, especially among the cereals and the *Brassicas*. Our strategy is

Copyright © 2001 John Wiley & Sons, Ltd.

to use rice to model potential orthology within cereals, using conserved linkage as a clue to possible ancestry, and use *Arabidopsis* as a model for *Brassicas*. *Arabidopsis* will not do for cereals because of the evolutionary distance, and the problem of ploidy levels is very complex, with rape having three copies of most *Arabidopsis* genes. It's very difficult to generalise if you don't have data on gene function in both compared species. With respect to transcriptional control, comparisons of promoter sequences between closely related species has been shown to reveal conserved sequences that may be regulatory. Comparisons between *Arabidopsis* ecotypes may reveal this, but one may have to go further, to *Capsella* (9–12 Myr distance) or *Brassicas* (15–20 Myr distance) to search for the appropriate level of sequence divergence needed to reveal conserved sequences of functional significance. *Arabidopsis* gene order is quite well conserved in *Brassicas*, and much less so in tomato.

**CFG: In your role as coordinator of EXOTIC, you are studying the expression pattern of around 5000 *Arabidopsis* genes. Will you be using microarrays? What strategies are you going to be using (e.g. will you be exposing the plants to a range of conditions)? How will this data be made available to the public?**

MB: In EXOTIC we will use gene trap expression data to assess gene function and expression in a wide range of conditions. At the JIC we are interested in a wide range of expression patterns, from those seen during specification of meristems, to stress responses. Ultimately, data generated here will probably be deposited in public databases, but that is a decision for the originators of the data.

**CFG: As a service provider for GARNet you are involved in a project to produce a large collection of transposon insertion lines and also to sequence the sites of insertion in these lines. Can you describe your approach? How will the data be distributed?**

MB: In GARNet we are making a population of 30,000 gene trap lines and will sequence the insertion sites of the transposons in all these lines. This sequence will be sent to Genbank and also integrated with the MIPS genome database. We are also making a new database called ATIS, which integrates genome sequence with insertion sites and expression patterns.

**CFG: Is there any follow up planned on this project in terms of phenotypic analyses, or will this be left to the wider *Arabidopsis* community? Are there any other experiments you would like to do with these lines?**

MB: The lines made in GARNet will be bulked and sent to the stock centres for distribution. We have no intention to screen systematically for phenotypes, but if funding permits, we would like to screen for GUS expression patterns, to extend the scope of work in the EXOTIC EC project.

**CFG: The *Arabidopsis* genome has a bigger proportion of genes in large gene families than *C. elegans*, or *Drosophila*, which could imply increased redundancy. Do you expect that most *Arabidopsis* genes will have a phenotype, or that it will be more similar to the case in yeast, in which many genes have no discernible phenotype under a range of conditions?**

MB: Previously we have been involved in a project to define the functions of MYB transcription factors in *Arabidopsis*, and identified disruptions in 32 genes (Meissner *et al.*, 1999). While phenotype screening is not yet completed, it seems that the closer you look, and the more you think about what to look for, coupled to good luck, will generally give you a phenotype. Well-established cases of highly overlapping gene functions in *Arabidopsis* are few, with the Shatterproof genes being the best example (Liljegren *et al.*, 2000).

**CFG: As coordinator of the European Union *Arabidopsis* Genome Sequencing Consortium you will have seen at first hand the excitement and motivation the genome sequence has given to the *Arabidopsis* community, would you like to comment on that and what has been achieved?**

MB: I think the *Arabidopsis*, and indeed the wider plant community, were pleased to have the sequence finished relatively quickly, with an unprecedented completeness and accuracy (The *Arabidopsis* Genome Initiative, 2000). The sequence has been thoroughly analysed by MIPS and TIGR and made available to the public in comprehensive databases. The bioinformaticians, sequencers and mappers did a fantastically thorough job against tight deadlines, and it was both a privilege and

Copyright © 2001 John Wiley & Sons, Ltd.

enormously exciting to work with these groups. Although there was a parallel industrial effort to sequence *Arabidopsis*, we were able to publish jointly with them, and their data is a potent source of polymorphisms for mapping. Like most scientific communities studying model organisms, the *Arabidopsis* genome project was generally characterised by openness and mutual support, and all but a few scientists respected the unspoken right of the sequencers to publish initial analyses of the whole genome sequence they had produced. The genome sequence can be used in a wide range of applications, and its final value won't be realized for many years. One particularly exciting development is the power of comparison between genomes – *Arabidopsis* and *Synechocystis* have many genes in common, and so do humans and *Arabidopsis*.

**CFG: You were also involved in organising the recent *Arabidopsis* Functional Genomics workshop at Cold Spring Harbor. What progress was made there towards defining the goals for *Arabidopsis* Functional Genomics studies? Do you think there will be similar international initiatives and consortia as for the sequencing? Was there any discussion of data standards or ontology issues?**

MB: The sequencing work was relatively closely organised, and I don't think at this stage functional genomics activities in *Arabidopsis* need to be as highly regulated. Gene disruptions are made by random insertions, so the first goal is to just get enough lines (primarily T-DNA) to yield a high probability of finding a disruption, then making these lines available through screening and ultimately sequencing the insertion sites, and integrating the data into public databases. This is all ongoing. Coordination is required in the integration of array analysis data from different labs, and this is also in hand. Beefing up the stock centres to handle the vast number of lines is a very high priority, and is needed if the gene disruption lines are to be readily available to users.

Various approaches are being taken to derive general descriptions of gene function. Presently the *Arabidopsis* functional catalogue consists of yeast functional categories, which have proved to be remarkably adaptable. Nevertheless, ontologies based on *Drosophila* gene descriptions are being developed, and there are also more novel approaches being considered. This work will continue to require a good deal of input from the community to devise standards and capture the varieties of descriptions of gene function.

### Related Websites

Arabidopsis Transposon Insertion Service (ATIS)
**http://www.jic.bbsrc.ac.uk/STAFF/michael-bevan/ATIS/**

Cold Spring Harbor *Arabidopsis* Genetrap Database
**http://spot.cshl.org/genetrap_database/mainframe.html**

Genomic *Arabidopsis* Resource Network (GARNet)
**http://garnet.arabidopsis.org.uk**

MIPS *Arabidopsis thaliana* database (MATDB)
**http://websvr.mips.biochem.mpg.de/proj/thal/**

The TIGR *Arabidopsis thaliana* Database
**http://www.tigr.org/tdb/at/at.html**

UK CropNet (UK Crop Plant Bioinformatics Network)
**http://ukcrop.net**

### References

Grant D, Cregan P, Shoemaker RC. 2000. Genome organization in dicots: Genome duplication in *Arabidopsis* and synteny between soybean and *Arabidopsis*. *Proc Natl Acad Sci U S A* **97**: 4168–4173.

Ku HM, Vision T, Liu JP, *et al.* 2000. Comparing sequenced segments of the tomato and *Arabidopsis* genomes: Large-scale duplication followed by selective gene loss creates a network of synteny. *Proc Natl Acad Sci U S A* **97**: 9121–9126.

Liljegren SJ, Ditta GS, Eshed HY, Savidge B, Bowman JL, Yanofsky MF. 2000. Shatterproof MADS-box genes control seed dispersal in *Arabidopsis*. *Nature* **404**: 766–770.

Maleck K, Levine A, Eulgem T, *et al.* 2000. The transcriptome of *Arabidopsis thaliana* during systemic acquired resistance. *Nature Genetics* **26**: 403–410.

Meissner RC, Jin HL, Cominelli E, *et al.* 1999. Function search in a large transcription factor gene family in *Arabidopsis*: Assessing the potential of reverse genetics to identify insertional mutations in R2R3 MYB genes. *Plant Cell* **11**: 1827–1840.

Sundaresan V, Springer P, Volpe T, *et al.* 1995. Patterns of gene-action in plant development revealed by enhancer trap and gene trap transposable elements. *Gene Dev* **9**: 1797–1810.

The Arabidopsis Genome Initiative. 2000. Analysis of the genome sequence of the flowering plant *Arabidopsis thaliana*. *Nature* **408**: 796–815.

Copyright © 2001 John Wiley & Sons, Ltd.

The Interview articles of *Comparative and Functional Genomics* aim to present a commentary on topical issues in genomics studies. They are a personal critical analysis, by the interviewee, of current work in their field of expertise, and aim at providing implications for future genomics studies. This interview was undertaken by the Managing Editor, Dr Joanne Wixon.

**Professor Michael Bevan**
*John Innes Centre, Norwich Research Park, Colney, Norwich, Norfolk, NR4 7UH, UK.*
E-mail: michael.bevan@bbsrc.ac.uk

The Genomics Website at Wiley www.wiley.co.uk/genomics

The **Genomics** website at Wiley is a **DYNAMIC** resource for the genomics community, offering **FREE** special feature articles and new information **EACH MONTH.**

Find out more about *Comparative and Functional Genomics,* and *Proteomics,* and how to view many articles **FREE OF CHARGE**!

Visit the **Library** for hot books in Genomics, Bioinformatics, Molecular Genetics and more.

Click on **Journals** for information on all our up-to-the minute journals, including: *Genesis, Bioessays, Gene Function and Disease, Human Mutation, Genes, Chromosomes and Cancer* and the *Journal of Gene Medicine.*

Let the **Genomics** website at Wiley be your **guide** to genomics-related web sites, manufacturers and suppliers, and a calendar of conferences.

WILEY

2171

Copyright © 2001 John Wiley & Sons, Ltd. 
